# Multiple Targeting Routes of MicroRNA-22 Inform Cancer Drug Discovery

**Published:** 2015

**Authors:** Jianhua Xiong

**Affiliations:** Center for Molecular Medicine, National Heart, Lung and Blood Institute, National Institutes of Health, Bethesda, Maryland, USA

MicroRNAs (miRNAs) are a class of ~23-nt endogenous non-coding RNA transcripts that mediate the posttranscriptional repression of target protein-coding genes [1]. Although the first evidence for the role of miRNAs in cancer development was presented in 2002 [2], the clinical trials of miRNA-based cancer drugs is just burgeoning recently [3]. Certain miRNA has been shown to be capable of targeting multiple mRNAs and affecting extensive protein output [4,5]. Particularly, miRNA-22 (miR-22) is one of the most studied multitasking miRNAs in tumor initiation and progression to date and attracts special attention from basic and clinical research scientists [6].

The first target estrogen receptor alpha (ERα) of miR-22 as a tumor suppressor was identified in breast cancer cells and clinical samples [7,8]. Subsequent studies reveal a series of oncogenic targets of miR-22 including HDAC4, SIRT1, p21, HIF-1α, EVI1, CDK6, EZR, Sp1, Wnt-1, TIAM1, MMP-2, MMP-9, CD151, CCNA2, PAPST1, KDM3A, BTG1, MTDH and CD147, MYCBP and MAX [6,9–19]. The expression of miR-22 could serve as a pivotal biomarker in tumor diagnosis and prognosis [6,20]. The tumor-suppressive function of miR-22 has been intensively investigated in various types of tumor cells and tissues including leukemia, lymphoma, endometrial endometrioid carcinoma, hepatoma, medulloblastoma, Ewing Sarcoma, and breast, lung, ovarian, colon, prostate, liver, gastric, cervical, colorectal and pancreatic cancer [6,9–19]. Of note, a regulatory loop involving miR-22 and robust oncogenic transcription factor c-Myc plays critical roles in breast tumorigenesis [18,19].

Contrary to the prevailing view of miR-22 as a tumor suppressor, the oncogenic effects of miR-22 has been characterized in leukemia, prostate cancer, cervical cancer,breast cancer, and bronchial epithelial cancer cells via PTEN signaling [6,21]. In addition, miR-22 can control cell stemness to promote hematopoietic transformation and breast cancer metastasis by directly targeting TET family members [22,23]. Upregulation of endogenous miR-22 expression can also cause impaired genomic integrity and DNA repair through inhibition of MDC1 in bone osteosarcoma cells [24]. Combined with these studies, cell stemness has been recently linked to low DNA damage implying the potential roles of miR-22 in coordinating cancer cell stemness and genomic stability [25,26]. Taken together, the opposing tumor suppressive and oncogenic activities of miR-22 may be two sides of the same coin, and they are both of critical importance in tumor cell homeostasis.

Owing to the advances in the miRNA-based therapeutics, chemically modified miRNA mimics and miRNA expression vectors hold great promise in tumor treatment and have already been in the clinical trials or developmental pipelines of pharmaceutical companies [6]. Although the cellular context is not a significant master of the majority of miRNA targets [27], it remains necessary to dissect the decisive factors for the multitasking of the evolutionarily conserved miR-22 [28]. Accumulating knowledge on the functional complexity and therapeutic safety will definitely improve the specificity and efficacy of miR-22-based tumor treatment. Previous studies in the quest to identify miR-22 functions have opened a new avenue for tumor treatment. Although there are numerous challenges to overcome for implementation of miRNA-based cancer therapy, it is expected that miR-22-based cancer drug will finally enter into clinical use and miRNA-associated treatment in patients with cancer will come of age.
